# A Deep Sequencing Approach to Comparatively Analyze the Transcriptome of Lifecycle Stages of the Filarial Worm, *Brugia malayi*

**DOI:** 10.1371/journal.pntd.0001409

**Published:** 2011-12-13

**Authors:** Young-Jun Choi, Elodie Ghedin, Matthew Berriman, Jacqueline McQuillan, Nancy Holroyd, George F. Mayhew, Bruce M. Christensen, Michelle L. Michalski

**Affiliations:** 1 Department of Pathobiological Sciences, University of Wisconsin-Madison, Madison, Wisconsin, United States of America; 2 Department of Computational and Systems Biology, Center for Vaccine Research, University of Pittsburgh School of Medicine, Pittsburgh, Pennsylvania, United States of America; 3 The Sanger Institute, Wellcome Trust Genome Campus, Hinxton, Cambridge, United Kingdom; 4 Department of Biology and Microbiology, University of Wisconsin Oshkosh, Oshkosh, Wisconsin, United States of America; Lindsley F. Kimball Research Institute - New York Blood Center, United States of America

## Abstract

**Background:**

Developing intervention strategies for the control of parasitic nematodes continues to be a significant challenge. Genomic and post-genomic approaches play an increasingly important role for providing fundamental molecular information about these parasites, thus enhancing basic as well as translational research. Here we report a comprehensive genome-wide survey of the developmental transcriptome of the human filarial parasite *Brugia malayi*.

**Methodology/Principal Findings:**

Using deep sequencing, we profiled the transcriptome of eggs and embryos, immature (≤3 days of age) and mature microfilariae (MF), third- and fourth-stage larvae (L3 and L4), and adult male and female worms. Comparative analysis across these stages provided a detailed overview of the molecular repertoires that define and differentiate distinct lifecycle stages of the parasite. Genome-wide assessment of the overall transcriptional variability indicated that the cuticle collagen family and those implicated in molting exhibit noticeably dynamic stage-dependent patterns. Of particular interest was the identification of genes displaying sex-biased or germline-enriched profiles due to their potential involvement in reproductive processes. The study also revealed discrete transcriptional changes during larval development, namely those accompanying the maturation of MF and the L3 to L4 transition that are vital in establishing successful infection in mosquito vectors and vertebrate hosts, respectively.

**Conclusions/Significance:**

Characterization of the transcriptional program of the parasite's lifecycle is an important step toward understanding the developmental processes required for the infectious cycle. We find that the transcriptional program has a number of stage-specific pathways activated during worm development. In addition to advancing our understanding of transcriptome dynamics, these data will aid in the study of genome structure and organization by facilitating the identification of novel transcribed elements and splice variants.

## Author Summary

Lymphatic filariasis, also known as elephantiasis, is a tropical disease affecting over 120 million people worldwide. More than 40 million people live with painful, disfiguring symptoms that can cause severe debilitation and social stigma. The disease is caused by infection with thread-like filarial nematodes (roundworms) that have a complex parasitic lifecycle involving both human and mosquito hosts. In the study, the authors profiled the transcriptome (the set of genes transcribed into messenger RNA rather than all of those in the genome) of the human filarial worm *Brugia malayi* in different lifecyle stages using deep sequencing technology. The analysis revealed major transitions in RNA expression from eggs through larval stages to adults. Using statistical approaches, the authors identified groups of genes with distinct life stage dependent transcriptional patterns, with particular emphasis on genes displaying sex-biased or germline-enriched patterns and those displaying significant changes during larval development. This study presents a first comprehensive analysis of the lifecycle transcriptome of *B. malayi*, providing fundamental molecular information that should help researchers better understand parasite biology and could provide clues for the development of more effective interventions.

## Introduction

*Wuchereria bancrofti*, *Brugia malayi* and *Brugia timori* are mosquito-borne filarial nematode parasites that cause the tropical disease lymphatic filariasis (LF). The manifestation of the disease ranges from swelling of the lymph nodes to elephantiasis and hydrocele. LF is a major cause of clinical morbidity and disability, leading to significant psychosocial and psychosexual burden in endemic countries. *B. malayi* is the primary organism for the study of LF because it has a tractable lifecycle that can be replicated in a laboratory setting. Like other filarial nematodes it is a heteroxenous parasite alternating between arthropod vectors and vertebrate hosts. Filarial nematodes are dioecious and reproduce sexually via copulation. Inseminated adult female worms are ovoviviparous and release live larvae (microfilariae) into the lymph, where they eventually circulate in the bloodstream to be taken up by mosquitoes during blood feeding. After a microfilaria (MF) successfully penetrates the midgut of a susceptible vector, it migrates to the thoracic muscles, and develops intracellularly through two molts to achieve the developmentally arrested third-stage larva (L3) that exits the mosquito proboscis during bloodfeeding and subsequently penetrates the mammalian host. Once L3s enter the definitive host, they undergo two additional molts and mature to adults in the lymphatics.

Characterization of the transcriptional program over the complete lifecycle is necessary to clearly understand the development of the parasite and could help devise better target strategies for control. From the standpoint of possibly designing drug-based or vaccine interventions that prevent infection or curtail parasite transmission, there is particular interest in understanding the biology of the L3 to L4 transition in the mammalian host, and the reproductive biology of filarial worms. The completion of the draft genome of *B. malayi* [1] has ushered in the possibility to use whole-genome gene expression profiling. With that goal in mind, we used next-generation sequencing to comparatively analyze the transcriptome of seven *B. malayi* lifecycle stages: eggs & embryos, immature MF (of less than 3 days of age), mature MF, L3, L4, adult male and adult female. We find that the transcriptional program has a number of stage-specific pathways activated during worm development and that a number of these are potential targets for drugs or vaccines.

## Methods

### Ethics statement

All animal work was conducted according to relevant national and international guidelines outlined by the National Institutes of Health Office of Laboratory Animal Welfare, and was approved under UWO Institutional Animal Care and Use Protocol 0-03-0026-000246-4-6-11; and UWM Research Animal Resource Center Protocol V00846-0-10-09.

### Parasites

*Brugia malayi* adults and MF were obtained from the peritoneal cavities of patently infected dark-clawed Mongolian gerbils (*Meriones uguiculatus*) by peritoneal flush with prewarmed RPMI media (Fisher Scientific, Piscataway, NJ). MF were purified by centrifugation through Ficoll-Paque® lymphocyte isolation media (Amersham Pharmacia Biotech, Piscataway, NJ), and washed in PBS three times prior to flash freezing at −80°C. Adult worms were separated by gender, washed three times in RPMI, and flash frozen. Egg and embryo preparations were made by repeated cutting of 10 female worms with a scalpel to release eggs and embryos into a small volume of cold RPMI. The sample was examined microscopically and pieces of uterine tissue were removed using watchmaker's forceps. The sample was washed three times in cold RPMI prior to flash freezing. Immature MF (≤3 days old) were generated and purified as previously described [2]. L4s were isolated from gerbils 12–13 days post peritoneal infection and were processed as described for adult worms. L3s were obtained from the NIAID-NIH Filariasis Research Reagent Resource Center at University of Georgia, Athens, GA.

### RNA isolation

Total RNA was isolated from the majority of samples using a previously described protocol [2] that combines organic extraction with Trizol LS (Invitrogen, Carlsbad, CA) and column purification (RNAqeous-Micro®, Applied Biosystems, Foster City, CA). Samples were treated with DNase I (Ambion, Austin, TX, USA) according to the manufacturer's instructions, and the absence of background DNA confirmed by using a portion of each sample in a PCR designed to amplify the *B. malayi* GPX gene [GenBank:X69128] (data not shown). Isolation of RNA from L3s often produces low yields therefore we used a modified protocol employing homogenization of tissue combined with organic extraction in RNAzol [3] followed by cleaning, concentration and DNase treatment using a Zymo Research RNA column (Zymo Research Corp, Orange, CA). For all samples RNA integrity was confirmed visually by agarose gel electrophoresis (data not shown) and purity and concentration determined spectrophotometrically (NanoDrop ND-1000, ThermoFisher Scientific); samples were stored at −80°C. Total RNA was lyophilized under vacuum for transport on dry ice to the Wellcome Trust Sanger Institute Genome Facility.

### RNA library creation

Polyadenylated mRNA was purified from total RNA using oligo-dT dynabead selection followed by metal ion hydrolysis fragmentation with the Ambion RNA fragmentation kit. First strand synthesis, primed using random oligonucleotides, was followed by 2nd strand synthesis with RNaseH and DNApolI to produce double-stranded cDNA using the Illumina mRNA Seq kit. Template DNA fragments were end-repaired with T4 and Klenow DNA polymerases and blunt-ended with T4 polynucleotide kinase. A single 3′ adenosine was added to the repaired ends using Klenow exo- and dATP to reduce template concatemerization and adapter dimer formation, and to increase the efficiency of adapter ligation. Adapters (containing primer sites for sequencing) were then ligated and fragments size-selected (200–275 bp) by agarose gel electrophoresis. DNA was extracted using a Qiagen gel extraction kit protocol but with dissolution of gel slices at room temperature (rather than 50°C) to avoid heat induced bias. Libraries were then amplified by PCR to enrich for properly ligated template strands, to generate enough DNA, and to add primers for flowcell surface annealing. AMPure SPRI beads were used to purify amplified templates before quantification using an Agilent Bioanalyser chip and Kapa Illumina SYBR Fast qPCR kit.

### Sequencing

Libraries were denatured with 0.1 M sodium hydroxide and diluted to 6 pM in a hybridization buffer to allow the template strands to hybridize to adapters attached to the flowcell surface. Cluster amplification was performed on the Illumina cluster station or the Illumina cBOT using the V4 cluster generation kit following the manufacturer's protocol. A SYBRGreen QC was performed to measure cluster density and to determine whether to pass or fail the flowcell for sequencing. This was followed by linearization, blocking and hybridization of the R1 sequencing primer. The hybridized flowcells were loaded onto the Illumina Genome Analyser IIx for 54 cycles of sequencing-by-synthesis using Illumina's v4 or v5 SBS sequencing kit then, *in situ*, the linearization, blocking and hybridization step was repeated to regenerate clusters, release the 2nd strand for sequencing and to hybridize the R2 sequencing primer followed by another 54 cycles of sequencing to produce paired end reads. These steps were performed using proprietary reagents according to manufacturer's recommended protocol (https://icom.illumina.com/). Data were analyzed using the RTA1.6 or RTA1.8 Illumina pipeline and submitted to Array Express (http://www.ebi.ac.uk/arrayexpress/) under the accession number E-MTAB-811.

### QC Analysis

Each lane of Illumina sequence was assessed for quality based on %GC content, average base quality and Illumina adapter contamination. To assess the quality of the lane, the mean base quality at each base position in the read was computed over all reads from the lane. To assess %GC content of the reads a frequency distribution of values was plotted. For a single sample in a lane, a GC plot with a normal distribution around the expected GC for the organism would be expected. Any lanes containing a contamination could therefore be identified by the presence of multiple peaks in the %GC plot. To screen for adapter contamination, the sequence reads were aligned to the set of Illumina adapter sequences using BLAT v.34 with default parameters [4]. Any reads matching these sequences were reported as being contaminated with adapter sequence.

### Sequence alignment and transcript quantification

Sequence reads from each lifecycle stage were aligned to the genome assembly [GenBank:DS236884–DS264093] using TopHat v1.0.14, a splice junction mapper built upon the short read aligner Bowtie [5], [6]. The pipeline utilized exon records in the genome annotation [1] to build a set of known splice junctions for each gene model, complementing its *de novo* junction mapping algorithm. Default parameters were used except for the following: minimum intron length was set to 50; minimum isoform fraction filter was disabled; closure-search, coverage-search, microexon-search and butterfly-search were enabled for maximum sensitivity. The resulting alignment files were converted to BAM format and low quality alignments with mapping quality scores less than 5 were removed before downstream analyses [7], [8]. No replicate samples were sequenced and all data were combined per lifecycle stage. Reads aligned to exonic regions were enumerated for each gene model using the HTSeq package (v0.4.7) in Python (www-huber.embl.de/users/anders/HTSeq). Reads overlapping more than one gene model were counted as ambiguous with the mode parameter set as “union”. Following Mortazavi et al. [9], transcript abundance estimates were computed as RPKMs (Reads Per Kilobase of exon model per Million mapped reads) with the following modifications: (i) a set of paired-end reads were counted as one in compiling sequence counts to represent a single sampling event and (ii) TMM (trimmed mean of M)-normalized values were used in place of the nominal library size to account for compositional biases [10]. The correction factors for TMM-normalization (i.e., the weighted trimmed mean of M values to the reference) were calculated using the Bioconductor edgeR package [11]. The weights were from the delta method on binomial data, and the library whose upper quartile is closest to the mean upper quartile was used as the reference.

### Differential expression analysis

Differential expression analysis was performed in edgeR by fitting a negative binomial model to the sequence count data. Using the quantile-adjusted conditional maximum likelihood method, dispersion parameters were estimated for each gene as a measure of the overall stage-to-stage variability to facilitate between-gene comparisons. All hypothesis testing was carried out using exact test for the negative binomial distribution with a common dispersion term for all genes. P-values less than 0.01 were considered significant. Dispersion parameters were estimated directly from the count data for comparisons contrasting a single stage or two related stages relative to all other stages. For comparisons between pairs of lifecycle stages, a common dispersion value of 0.2 was used, which is equivalent to allowing within-stage variations in expression levels of up to 45%. This value was chosen based on the level of variability observed between the immature and mature MF samples. Because longer transcripts give more statistical power for detecting differential expression between samples [12], Gene Ontology (GO) analysis was performed using the goseq package that adjusts transcript length bias in deep sequencing data [13]. GO annotation was retrieved from the UniProtKB-GOA database [14], and statistically over-represented GO terms in a given gene list were identified using the Wallenius non-central hypergeometric distribution. Hierarchical clustering analysis was performed using GeneSpring GX (Agilent Technologies). RKPM values for each gene were baseline transformed to the median of all samples, and hierarchically clustered with centroid linkage using Pearson's uncentered correlation coefficient as distance metric.

## Results

### Transcriptome quantification by deep sequencing

**Figure 1:**
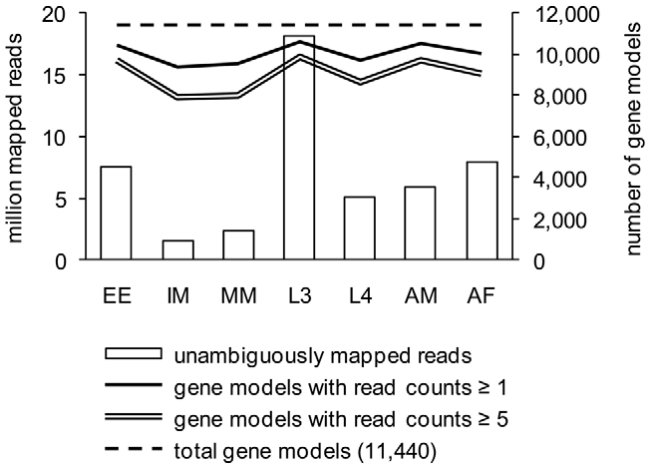
Library size and gene model coverage. Library size refers to the total number of reads unambiguously mapped to gene models. At the sequencing depth of the current study, ≥8,000 gene models had 5 or more mapped reads in each lifecycle stage library.

In total, 104 million paired-end reads (2×54 bp) were generated from polyA-tailed mRNA using the Illumina Genome Analyser IIx (Table S1). Sequence reads were aligned to the genome assembly using TopHat [5], and the number of reads aligned to each gene model was summed yielding relative transcript levels for individual genes. Approximately 50% of the sequenced reads were mapped to the reference genome after low quality alignments were removed; 10% of which were aligned to genomic regions outside of the current gene models. Sequencing depth varied between the lifecycle stage libraries, affecting gene model coverage and the distribution of the read counts per gene model for each library (Figure 1 and Figure S1). Overall, in each library, 8,000–10,000 genes (equivalent to 70 to 90% of the currently annotated gene models) had 5 or more mapped reads. Sequence counts were RPKM (Reads Per Kilobase of exon model per Million mapped reads)-transformed and TMM (trimmed mean of M)-normalized to assist in the interpretation of transcript abundance comparisons between stages and genes [9], [10]. For statistical inferences, however, raw read counts were directly used. Further analysis of our sequence data from a genomics perspective, covering issues related to missing, incomplete or incorrect gene models of the 2007 assembly [1] will be published elsewhere (in preparation).

Our sequencing libraries contained reads that map to the *Wolbachia* genome [GenBank:AE017321]. However, the study was not adequately designed such that one could quantitatively analyze these reads in a biologically meaningful way. Abundance estimates (inferred from read counts) of these transcripts most likely deviate substantially from their true *in vivo* levels. Poly-A selection directly affects the relative abundance of non-poly-A *Wolbachia* transcripts with respect to *B. malayi* transcripts. Moreover, the nature and extent of the biases introduced by oligo-dT method to the relative abundance levels among the non-poly-A species (with respect to each other) is not well understood, and one cannot assume that these biases would remain uniform among different sample preparations. Another layer of uncertainty stems from the possibility that these “*Wolbachia*” sequences were transcribed from the *B. malayi* nuclear genome rather than the endosymbiont as a consequence of the past horizontal gene transfer events, leading to a differential capture of (presumably) poly-A tailed “*Wolbachia*” transcripts of the *B. malayi* nuclear origin. However, given the incomplete draft nature of the *B. malayi* genome assembly and the inherent difficulty in mapping short reads originating from multiple loci that are similar in sequences, it remains challenging to rigorously test this hypothesis *in silico*.

### Lifecycle stage dependent changes in the transcriptome

**Figure 2:**
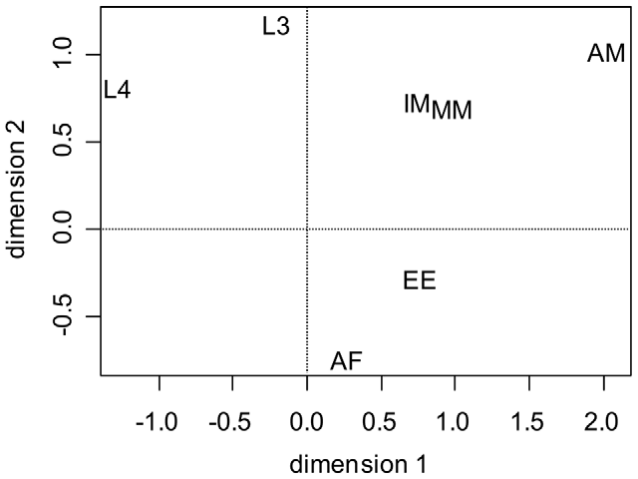
Multidimensional scaling (MDS) plot showing sample relations. The distance between each pair of samples was the square root of the common dispersion for the top 500 genes that best distinguished that pair of samples. These top 500 genes were selected according to the tagwise dispersion of all the samples.

To investigate the global transcriptional differences between stages and between genes during development, a negative binomial (NB) based model [11] was fit to sequence count data. First, the degree of between-stage differences was assessed globally using a multidimensional scaling (MDS) of all-against-all comparisons in the NB model (Figure 2). The resulting sample relations appear consistent with the expected biological differences between the samples. The MDS plot indicates that, in relative terms, the transcriptome profiles of the immature and mature MF are more similar to each other than either is to other stages. Likewise, the eggs & embryos sample is closely related to the adult female sample, part of which consists of the germ-line cells. Interestingly, this plot also shows how different the transcriptome profiles of adult male and female worms are to each other.

Next, we made between-gene comparisons in terms of overall transcriptional variability across stages. It is generally hypothesized that while some genes are expressed constitutively, genes with specific developmental functions are expressed at specific stages. To quantify the level of transcriptional variation for each gene across the seven lifecycle stages, the NB dispersion parameters were estimated for each gene, and used as a measure of the extra-Poisson, stage-to-stage variability. Genome-wide distribution of the dispersion parameter estimates suggests that the level of transcriptional variation is not uniform across all genes (Figure S2). Although the majority of genes show low to moderate levels of variation, certain groups of genes exhibit a significantly greater level of variation. Approximately 25% of genes have NB dispersion parameter values larger than 1. After ranking by dispersion, genes were partitioned into quarters and designated as Q1 through Q4 in the order of decreasing variability.

**Figure 3:**
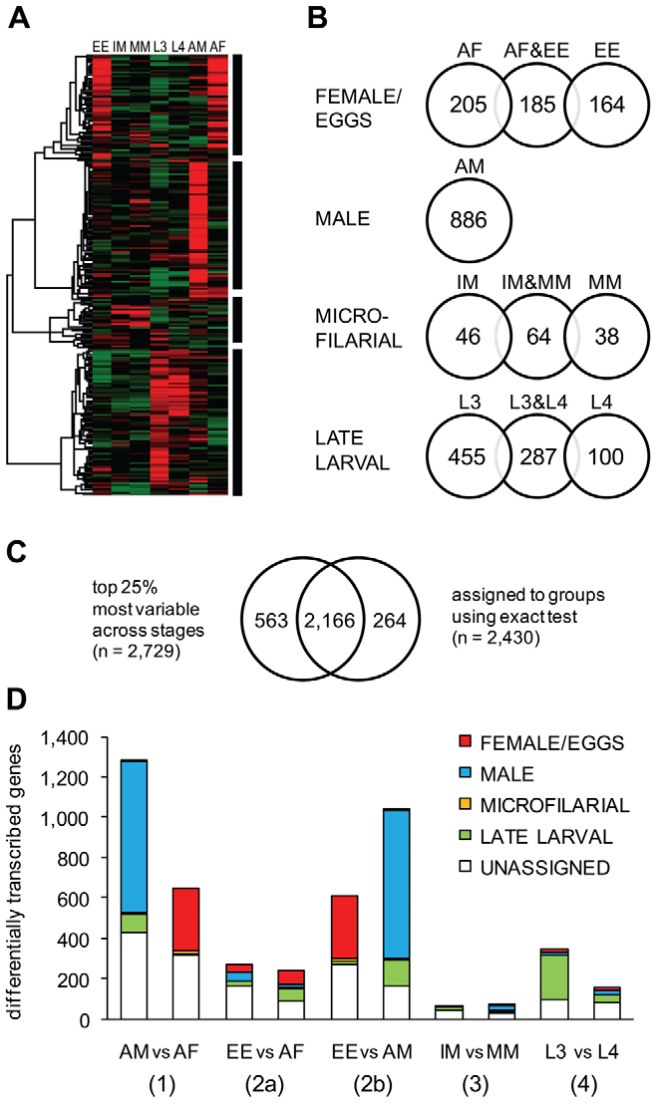
Comparison of transcriptome profiles between lifecycle stages. (A) Unsupervised hierarchical clustering of the RPKM values of the top 25% most variable genes according to the NB dispersion parameter (see Figure S2). (B) The number of genes classified into groups with distinct life stage dependent transcriptional patterns using a series of exact tests for the NB distribution (p-value<0.01). (C) Venn diagram showing that members of the expression groups identified through the exact tests account for ∼80% of the top 25% genes most variable in transcript abundance. (D) The number of differentially transcribed genes. Five direct pairwise comparisons were made between relevant stages to gain insights into the transcriptomic features associated with (1) sex differences, (2) intrauterine reproductive processes, (3) MF maturation, and (4) late larval development. Differentially transcribed genes were cross-referenced with the previously defined coexpression groups (Figure 3B).

To examine genes displaying life stage dependent transcriptional patterns in greater detail, the top 25% most variable genes according to the NB dispersion (i.e., Q1) were subjected to an unsupervised hierarchical clustering (Figure 3A). The resulting heatmap and dendrogram suggest that there are four major transcriptional patterns, each of which corresponds to an increased transcript abundance in (i) female and/or eggs & embryos, (ii) male, (iii) microfilariae, or (iv) late larval stages. The transcriptional patterns identified through the clustering analysis largely recapitulate the sample relations revealed in the MDS plot (Figure 2). To classify genes into these broad but distinct co-expression groups in a statistically robust manner, we performed a series of exact tests for the NB distribution using raw read counts for all genes (Figure 3B). Relying solely on the “shape” of expression patterns derived from RPKM values, without considering how many reads contributed to each pattern, may lead to false-positive findings. We first identified genes preferentially transcribed during single stages by performing exact tests contrasting each individual stage relative to the mean of all other stages. The resulting gene lists were augmented by additional exact tests to include genes displaying increased transcript abundance in two (related) stages with respect to all other stages. At the level of p-value<0.01, mutually-exclusive, non-redundant gene lists were compiled for each group. In total, we cataloged 2,430 genes into groups with distinct life stage dependent transcriptional patterns. Comparing the gene lists to the highly variable genes in the Q1 group suggests that members of the four main expression groups account for ∼80% of the top 25% most variable genes (Figure 3C). Genes that are highly variable in transcript abundance, yet are not assigned to any of the four main groups (n = 563) likely display complex transcriptional patterns falling outside of the four categories. In addition, five direct pairwise comparisons were made between relevant stages to gain further insights into the transcriptomic features associated with (1) sex differences, (2) intrauterine reproductive processes, (3) MF maturation, and (4) late larval development (Figure 3D). Cross-referencing with the previously defined coexpression groups (Figure 3B) indicates that stage specificity is not homogeneous within each group of differentially transcribed genes, highlighting the complexity of the relative transcriptome differences among the lifecycle stages examined in the study. The results outlined above are described in further detail in the following sections.

### Genes displaying high levels of transcriptional variation over lifecycle

We identified and compared statistically overrepresented GO terms in groups of genes that differ in their level of transcriptional variation over the lifecycle (i.e., Q1 to Q4) to investigate specific gene sets and functional categories distinctly associated with high levels of transcriptional variation (Table S2 and Figure S2). This analysis identified ‘structural constituent of cuticle’ (GO:0042302) as the most significantly overrepresented GO category among Q1 genes that exhibit high levels of between-stage transcriptional variation. Forty-six cuticle collagen genes are annotated with this GO term, and thirty-three of these have distinct lifecycle stage dependent transcriptional patterns (18 late larval, 12 female/eggs, 2 male and 1 microfilarial; Dataset S1). Additional GO terms overrepresented among Q1 genes include those related to serine type endopeptidase inhibitor (serpin), structural molecule, and kinase/phosphatase activity. By contrast, GO categories associated with protein metabolism, such as translation, protein transport and proteasome complex are significantly overrepresented among genes displaying relatively little transcriptional variation over lifecycle stages (i.e., Q2-4).

### Sex-biased and germline-enriched transcriptome

Although transcript levels of 990 genes are significantly higher during larval stages, 886 and 554 genes display elevated transcript abundance in adult male, and adult female and/or eggs & embryos, respectively (Figure 3B). A direct pairwise comparison of male versus female transcriptome further indentified 1,279 genes with male-biased expression and 651 genes with female-biased expression (Figure 3D). At the level of GO categories, structural molecular activity and those associated with protein phosphorylation and dephosphorylation are prominent among genes preferentially transcribed in adult male. A closer look at individual genes with male-biased expression reveals that major sperm proteins are largely responsible for driving the statistical significance of structural molecular activity (GO:0005198) in these comparisons. By contrast, structural constituents of cuticle (collagens), transcription factor/regulator activity, nuclear receptor activity and serpin activity constitute a main theme of the overrepresented functional categories among genes preferentially transcribed in adult female and/or eggs & embryos.

In an effort to elucidate female germline-enriched transcripts and gain insight into intrauterine reproductive processes, the transcriptome profile of a library enriched for eggs and embryos was compared with that of whole adult female (Figure 3D). However, because the eggs & embryos transcriptome is inherently a subset of the adult female transcriptome, this pairwise comparison is almost subtractive in nature and is likely biased against identifying transcripts enriched in germline tissues. On the contrary, detection of female transcripts either not expressed or expressed at lower levels in eggs and embryos likely remains unaffected by this asymmetric sample relation. For this reason, we used the adult male transcriptome profile as an additional reference point to better identify genes showing a germline-enriched expression pattern. We performed a Venn diagram analysis with three datasets: (1) genes with enriched expression in adult female relative to eggs & embryos, (2) genes with enriched expression in eggs & embryos relative to adult male, and (3) genes with enriched expression in adult female and/or eggs & embryos relative to all other stages (Figure S3). We considered genes belonging to the first set to exhibit somatic tissue-enriched expression pattern, and those belonging to either of the last two sets, but excluded from the first set, to exhibit germline-enriched expression pattern. Based on these criteria, 788 and 239 genes show enriched expression in female germline and somatic tissues, respectively. GO term overrepresentation analysis indicates that functional categories, such as transcription factor activity, DNA binding, regulation of transcription and nuclear receptor activity are more frequently found among genes displaying germline-enriched expression. On the contrary, genes implicated in chloride transport, lipid binding, and proteolysis are overrepresented among those with somatic tissue-enriched expression pattern (Table S3). Interestingly, structural constituents of cuticle (GO:0042302) is overrepresented among both genes with germline-enriched and somatic tissue-enriched expression patterns. A closer look at individual genes reveals that mutually exclusive subsets of collagens are overrepresented in each gene set.

### MF, L3 and L4 transcriptome

When compared across all stages, transcript levels of 148 genes are distinctly elevated during the MF stage. Overrepresented GO terms in this group include zinc ion binding, nucleic acid binding, chitinase activity, and proteolysis (Figure 3B and Table 1). Most notably, among these are 44 genes that encode proteins with C_2_H_2_-type zinc finger domains. There are 195 zinc finger protein genes annotated in the *B. malayi* draft genome, some of which have high transcript levels in stages other than MF (i.e., 3 late larval, 17 male and 6 female/eggs). In a similarly biased manner, 3 out of 4 endochitinase genes identified in the current *B. malayi* genome show transcriptional increase during MF stages. Diverse classes of proteases are also represented in this gene set (e.g., cathepsin L-like proteases including *Bm*-*cpl*-6, papain cysteine protease family, metalloprotease I, aspartyl protease and trypsin-like protease). Direct comparison of immature and mature MF (IM and MM) indicates that 126 genes show differential transcript abundance between the two samples (Figure 3D). Many different metabolic genes are found in the IM overexpressed gene set, while the endochitinases are overrepresented in the MM.

**Table 1:** List of over-represented GO terms in gene groups with distinct lifecycle stage dependent transcriptional patterns.

MICRO-FILARIAL	LATE LARVAL	MALE	FEMALE/EGGS	GO terms	
				**cellular component**	
1.8E-22				intracellular	GO:0005622
	1.2E-05			integral to membrane	GO:0016021
	2.1E-04			membrane	GO:0016020
	2.9E-04			intermediate filament	GO:0005882
		7.8E-05		cytoskeleton	GO:0005856
			6.2E-07	nucleus	GO:0005634
				**molecular function**	
1.2E-23				zinc ion binding	GO:0008270
1.4E-10				nucleic acid binding	GO:0003676
9.7E-06				chitinase activity	GO:0004568
1.6E-05				cation binding	GO:0043169
2.5E-05				hydrolase activity, acting on glycosyl bonds	GO:0016798
2.5E-05	1.8E-06			cysteine-type peptidase activity	GO:0008234
7.6E-04				hydrolase activity, hydrolyzing O-glycosyl compounds	GO:0004553
	9.2E-10		1.8E-06	structural constituent of cuticle	GO:0042302
	2.9E-06			cysteine-type endopeptidase activity	GO:0004197
	1.6E-05			transporter activity	GO:0005215
	3.3E-05			oxidoreductase activity	GO:0016491
	4.5E-05			extracellular matrix structural constituent	GO:0005201
	9.5E-05		3.8E-04	serine-type endopeptidase inhibitor activity	GO:0004867
	1.2E-04			voltage-gated chloride channel activity	GO:0005247
	4.7E-04			calcium ion binding	GO:0005509
	8.8E-04			hedgehog receptor activity	GO:0008158
		9.5E-18		phosphoprotein phosphatase activity	GO:0004721
		1.1E-13		protein kinase activity	GO:0004672
		6.7E-12		structural molecule activity	GO:0005198
		6.3E-10		kinase activity	GO:0016301
		2.8E-08		protein tyrosine phosphatase activity	GO:0004725
		1.0E-06		protein tyrosine kinase activity	GO:0004713
		5.7E-06		phosphatase activity	GO:0016791
		5.8E-04		ATP binding	GO:0005524
			1.6E-11	transcription factor activity	GO:0003700
			5.5E-10	sequence-specific DNA binding	GO:0043565
			4.4E-07	DNA binding	GO:0003677
			6.1E-05	ligand-dependent nuclear receptor activity	GO:0004879
			1.0E-04	transcription regulator activity	GO:0030528
				**biological process**	
1.0E-04	1.9E-04			proteolysis	GO:0006508
6.2E-04				chitin catabolic process	GO:0006032
	1.4E-06			metabolic process	GO:0008152
	6.0E-06			oxidation reduction	GO:0055114
	4.0E-05			transport	GO:0006810
	1.2E-04			chloride transport	GO:0006821
	3.2E-04			cell adhesion	GO:0007155
	5.8E-04			glycogen biosynthetic process	GO:0005978
		3.7E-13		protein amino acid phosphorylation	GO:0006468
		2.0E-07		protein amino acid dephosphorylation	GO:0006470
		1.7E-06		dephosphorylation	GO:0016311
			2.4E-10	regulation of transcription	GO:0045449
			5.3E-10	regulation of transcription, DNA-dependent	GO:0006355
			9.1E-05	transcription	GO:0006350

The gene groups used for the analysis were defined through a series of exact tests for NB distribution (Figure 3B). Over-represented GO terms with p-value<0.01 were included.

We identified 842 genes displaying increased transcript abundance during L3 and/or L4 stages relative to other lifecycle stages (Figure 3B). Functional categories overrepresented among these genes include structural components of the cuticle, oxidoreductase activity, serpin activity, chloride transport, hedgehog receptor activity, glycogen biosynthetic process, and proteolysis. As suggested by the last GO category, various proteases (e.g., metalloprotease, papain family peptidase, zinc carboxypeptidase family and cathepsin-like cysteine proteases, including *Bm*-*cpl*-1,4 and 5) are prominently represented in this gene set, a pattern similarly found in the MF transcriptome. A pairwise comparison of the transcriptomes of late larval stages indicates that 342 genes have elevated transcript levels in L3s, and 155 in L4s. At the level of functional categories, cysteine-type peptidase activity (e.g., cathepsin-z and -L like proteases) and serpin activity are overrepresented among L3-enriched transcripts, whereas structural constituents of the cuticle and cellular component organization are overrepresented among L4-enriched transcripts (Table S3). In addition, our data indicate that abundant larval transcripts (Alt1.2 and Alt2) show increased abundance in L3s relative to L4s.

## Discussion

Using high-throughput sequencing, we have undertaken a comprehensive genome-wide survey of the developmental transcriptome of the human filarial parasite *B. malayi*. Although deep sequencing data are highly informative in identifying novel transcribed elements and splice variants that help improve genome annotation [15], the present study aims to characterize transcriptome changes along the progression of the parasite's lifecycle. Transcriptome changes mediating cuticular molting likely represent one of the most notable developmental transitions in RNA expression. Like all nematodes, *Brugia* spp. have five lifecycle stages that are punctuated by molting of the collagenous cuticle. The tightly regulated process of molting involves cell signaling within the hypodermis to cue secretion of the new collagenous cuticle, shedding of the old cuticle and proteolytic remodeling of the new cuticle [16], [17]. Analysis of overrepresented GO terms highlights structural cuticle components, extracellular matrix components and cysteine-peptidase inhibitors, among others, in genes with high levels of transcriptional variation over the lifecycle (Table S2). In particular, the cuticle collagen gene family displays distinct dynamic transcriptional patterns over the course of the lifecycle, likely reflecting compositional variation in cuticular structure among different life stages. Besides these structural components, genes displaying the most dramatic transcriptional variation in our data set are likely associated with developmental processes that differ between the larval and the adult stages and/or between the genders (e.g., gametogenesis). By contrast, genes constitutively expressed over the developmental period studied frequently have predicted cellular functions related to protein expression, modification and transport, possibly representing core cellular processes that are essential to the survival of cells independent of the lifecycle stage.

The present study indicates that genes exhibiting adult male enriched transcriptional pattern (relative to adult female and/or other stages) show strong statistical bias towards GO categories related to cytoskeleton, structural molecule activity, protein phosphorylation and dephosphorylation (Table 1). Many of these gene sets and functional categories are highly represented among classes of male-enriched transcripts in parasitic nematodes [18], [19], [20], [21] and have been identified in the *Caenorhabditis elegans* male and hermaphrodite germline as being involved in spermatogenesis [22]. Nematode sperm are unique in that they utilize a nematode-specific cytoskeletal element, major sperm protein, for ameboid motility. It is hypothesized that because mature nematode sperm lack ribosomal elements, the phosphorylation and dephosphorylation of molecules by a host of enzymes within the differentiated cells could promote maturation and pseudopod extension [22]. Seven of the genes found to be differentially expressed in male worms in our study were also found in a microarray comparison of adult male and female worms [23], and were shown by *in situ* localization to be expressed either in sperm or vas deferens tissue of adult male worms and not in gravid adult female worms [24]. If we compare our RNA-seq data with recent microarray work comparing gene expression in adult male and female *B. malayi* [19], 515 of our 1,276 (40%) genes with male-biased expression match with male up-regulated genes found in the microarray comparison, and 150 out of the 651 (23%) genes with female-biased expression match the microarray findings.

In filarial nematodes, fertilization is internal and gravid females hold oocytes, sperm, zygotes, developing embryos, and MF in their uteri. Structural constituents of cuticle, transcription factor activity, DNA binding, and regulation of transcription emerged as notable themes in our analysis of overrepresented functional categories among genes with increased transcript levels in adult female and/or eggs & embryos (Table 1). These are likely relevant in the context of embryogenesis. Pairwise comparison of adult female with adult male presents us with a similar but more expanded view on features of genes displaying female-enriched expression (Table S3). Further comparisons with genes displaying germline-enriched expression patterns suggest that many of the female-biased transcripts, and more importantly, the majority of the above mentioned functional categories are attributable to the characteristics of the germline transcriptome. For instance, 33 out of 34 genes annotated with transcription factor activity (e.g., nuclear hormone receptors and homeobox domain containing proteins) that are enriched in female and/or eggs & embryos, have a distinctly germline-enriched expression pattern. Bm-*fab*-1 (Bm1_33050), an embryonic fatty acid binding protein transcript previously found to be female-associated by differential display PCR and whose protein localizes to embryos [25], [26] also exhibits a germline-enriched expression pattern.

Much of our current information on molecular aspects of filarial reproduction comes from microarray and PCR-based transcriptome comparisons between whole adult male and female worms. These studies were based on the assumption that gender-associated transcripts arise from the reproductive organs and their contents. Our data suggest that such an assumption is not wholly unreasonable but may not always hold true. Out of 651 female-enriched transcripts we identified (in comparison to male), 82 display somatic tissue-enriched expression patterns, and it is likely that some of these transcripts are truly not derived from the germline tissues. Spatial expression patterns have not been confirmed for the majority of gender-associated *B. malayi* genes, and a growing body of research on nematode neurobiology and extracellular signaling lends support to the idea that some gender-associated genes can be expressed in non-reproductive tissues. For example, free-living and parasitic nematodes use gender-specific receptors to sense environmental signals, as demonstrated by the presence of anterior chemosensors in male worms that specifically bind female pheromones [27], [28]. Nematodes also store fat in intestinal cells, which may act as endocrine organs involved in germline signaling and are triggered by activation of intestinal cell nuclear receptors by lipophilic hormones [29], [30], [31].

On the other hand, these observations are not inconsistent with the possibility that some somatic tissue derived transcripts play an essential role in embryonic development or intrauterine reproductive processes. The current study suggests that components incorporated into the embryonic cuticle and the eggshell membrane may be in some part maternal in origin. This interpretation is supported in at least one case where MF sheath protein transcripts in *Brugia* are detectable by *in situ* hybridization only in adult female tissues and not in eggs or embryos [24], while the encoded protein is found on the surface of *in utero* sheathed MF but not in maternal tissues [32]. Other notable transcripts showing enrichment in female somatic tissues in our study include Juv-p120 excretory/secretory proteins and astacin proteases (Bm1_30065; Bm1_13915). Homologs of the latter in *C. elegans*, *nas-4* and *nas-9* are found in pharyngeal marginal cells, and in the hypodermis and reproductive tract, respectively [33]. Their functions are unknown but the localizations suggest roles in cuticle and eggshell remodeling.

After expulsion from females, developmentally arrested *Brugia* MF must undergo a maturation process within the mammalian host to become infective to the mosquito vector [2], [34], [35]. *Brugia* MF are sheathed in a remnant of the eggshell membrane that is acellular and insoluble, and is composed of chitin and a variety of cross-linking proteins, lipids and polysaccharides [36], [37], [38], [39]. Our data indicate that a large number of transcripts representing DNA-binding proteins with zinc finger motifs as well as several endochitinase transcripts are significantly elevated in MF. Proteomic analysis also revealed a significant enrichment of zinc finger proteins in this stage of the lifecycle [21]. Although the precise role of these DNA binding proteins is unknown, it is tempting to speculate on their involvement in maintaining the developmentally arrested state of circulating MF. Transcriptional increase in proteases and chitin-associated enzymes in MF could be important in the process of casting off the chitinous sheath during or after mosquito midgut penetration [35], [40], [41]. Immunolocalization studies have shown that in sheathed MF, chitinase is stored in the inner body of the MF and secreted to the surface to degrade the sheath upon mosquito infection [42]. Microfilarial maturation is accompanied by transcriptional transitions and changes in the composition of the microfilarial surface [2], [35]. Despite the remarkable change in infectivity, our data suggest that transcriptional differences between IM and MM are relatively small; it is the least pronounced of all pairwise comparisons made in this study (Figure 2 and 3D). Genes involved in ATP synthase activity, tRNA production and cytoskeleton are overrepresented among those that show transcriptional change between IM and MM (Table S3). Although it is difficult to further characterize the exact nature of these changes due to a high proportion of genes with no functional annotation, we hypothesize that a metabolic shift is likely part of the maturation process in anticipation of the transition from the blood of a homeothermic host to the inhospitable midgut and hemocoel of the poikilothermic mosquito vector. It is important to consider that both populations of MF used in this experiment were derived from the peritoneal cavities of infected gerbils. Although we have previously shown a dramatic difference in mosquito infectivity between peritoneally-derived immature and mature MF [2], [34], [35], it is clear that intraperitoneally-derived MF, regardless of age, are considerably less infective than those found in circulating blood [43]. It is possible that the transcriptional profile of mature circulating MF differs from those that are derived from the peritoneal cavity.

Following the introduction of L3s into the peritoneal cavity of gerbils, the L3 to L4 transition requires no migration and occurs approximately 8 days post infection (unpublished). This particular lifecycle transition is of great interest to researchers trying to identify parasite molecules that mediate interactions with the host immune system, and that could be exploited with vaccines to confer protective immunity, or with drugs to prevent infection. Antigens that historically have been of interest in this regard are the ALT (abundant larval transcript) family of potentially secreted larval acidic proteins found predominately in L2 and L3 stages [44], [45], [46]; the L3 cystatin cysteine protease inhibitor family, Bm-SPN-2, TGF-β homologues, macrophage inhibition factor and Bm-VAL-1 [46]; troponin, tropomyosin and cuticular collagens [47]; *Onchocerca volvulus* activation associated secreted protein (Ov-ASP-1) [48], onchocystatin (Ov-CPI-2) [49] and Ov-SPI-1 [50], and *B. malayi* glutathione-s-transferase [51]. One hypothetical protein found to be L3 specific in our experiment, Bm1_38105, was also highly ranked as a potential drug target [52].

In the present study, the transcriptome of developmentally arrested, vector-derived L3s was compared to that of peritoneally-derived L4s at 12–13 days post infection. Comparing our RNA-seq data to a recent microarray experiment [53] that assessed transcriptomes of vector-derived L3s to cultured and irradiated L3s, shows that 29 genes are shared, and likely constitute genes required for L3 survival in mosquitoes. These include Alt-2 and Alt1.2 proteins, cathepsin L precursors, Bm-*col*-2, cystatin, microfilarial surface associated protein, metabolic proteins and BmSERPIN. The differential expression of cathepsins *Bm*-*cpl*-1, 4, and 5 in vector stage L3s is supported by EST sequences and these genes are grouped phylogenetically into a distinct clade (Ia) separate from other nematode cathepsin-like proteases [54]. There is strong evidence that these proteins play important roles in the L3 to L4 molt, because targeting the *cpl*-1 gene in *O. volvulus* by RNAi decreased the rate of molting [55], and suppression of the cathepsin L-like cysteine protease transcript by injection of siRNA or dsRNA into infected mosquitoes carrying L2 and L3 stages of *B. malayi* retarded worm growth, disrupted development and resulted in cuticular sloughing [56]. It is important to point out that the L4s we used were from the peritoneal cavity of gerbils, and did not follow the normal behavioral pathway of intradermal passage and migration to the lymphatics. It is possible that the transcriptional profile of intraperitoneally-derived L4s is different than that of worms found in lymphatics; indeed Chirgwin et al. [57] showed different transcriptional profiles for three L3 genes at 3 days post infection in groups that had been injected intradermally and allowed to migrate naturally to the popliteal lymph node in the gerbil model, and those that were confined to the peritoneum.

In this study we provide a detailed overview of the molecular repertoires that define and differentiate distinct lifecycle stages of the parasite, extending and complementing previously published work on stage-specific gene expression [2], [19], [21], [24], [53], [58], [59]. Inclusion of seven different developmental stage samples uniquely allows us to place specific between-stage transcriptional differences into the broader context of the transcriptomic landscape during the lifecycle of *B. malayi*. It is important to emphasize, however, that this is just an overview of observations and that these data will be mined by the community to provide specific information on particular gene sets to bring these deep sequencing data into more complete biological context.

Because expression dynamics is an important consideration in the genome-wide assessment of candidate targets for control [52], [60], [61], our comprehensive analysis of transcript abundance over developmental time is a valuable addition to a growing body of genomic and post-genomic resources that guide and support the concerted efforts to develop better intervention strategies.

## Supporting Information

Figure S1 Histogram of raw read counts per gene model and scaling-normalized RPKM values. To facilitate transcript abundance comparisons between genes and stages, read counts were RPKM-transformed and TMM-normalized [9], [10]. The distribution of transcript level estimates indicated 4 to 5 logs of dynamic range.
(PDF)

Figure S2 Genome-wide distribution of the dispersion parameter estimating stage-to-stage variability in transcript abundance. Based on the NB dispersion parameter, genes were ordered from the most variable to the least variable, and partitioned into four equal-sized groups as indicated by the horizontal dotted lines. GO term enrichment tests were performed on each of the four groups (Table S2).
(PDF)

Figure S3 Venn diagram analysis to identify genes with female somatic tissue- or germline-enriched expression pattern. (1) genes with enriched expression in adult female relative to eggs & embryos; (2) genes with enriched expression in eggs & embryos relative to adult male; (3) genes with enriched expression in adult female and/or eggs & embryos relative to all other stages.
(PDF)

Table S1 Total number of reads sequenced and mapped to the genome.
(PDF)

Table S2 List of over-represented GO terms in groups of genes with different levels of overall variability in their transcript abundance across lifecycle stages. Genes were partitioned into four equal-sized groups designated as Q1 to 4 in the order of decreasing variability (Figure S2). Over-represented GO terms with p-value<0.01 were included.
(PDF)

Table S3 List of over-represented GO terms and their corresponding p-values among genes that show relative transcriptional enrichment in pairwise comparisons (Figure 3D). Female somatic tissue- and germiline-enriched patterns were defined using Venn diagram analysis (Figure S3).
(PDF)

Dataset S1 Gene-level RNA-seq read counts and RPKM values for *Brugia malayi* lifecycle transcriptome.
(XLS)
