# Supplementary material for: A Deep Sequencing Approach to Comparatively Analyze the Transcriptome of Lifecycle Stages of the Filarial Worm, Brugia malayi
Source: PLoS Negl Trop Dis. 2011 Dec 13;5(12):e1409. doi: 10.1371/journal.pntd.0001409 (PMC3236722; doi:10.1371/journal.pntd.0001409)

Figure S1. Histogram of raw read counts per gene model and scaling-normalized RPKM values. To facilitate transcript abundance comparisons between genes and stages, read counts were RPKM-transformed and TMM-normalized (see Methods). The distribution of transcript level estimates indicated 4 to 5 logs of dynamic range.

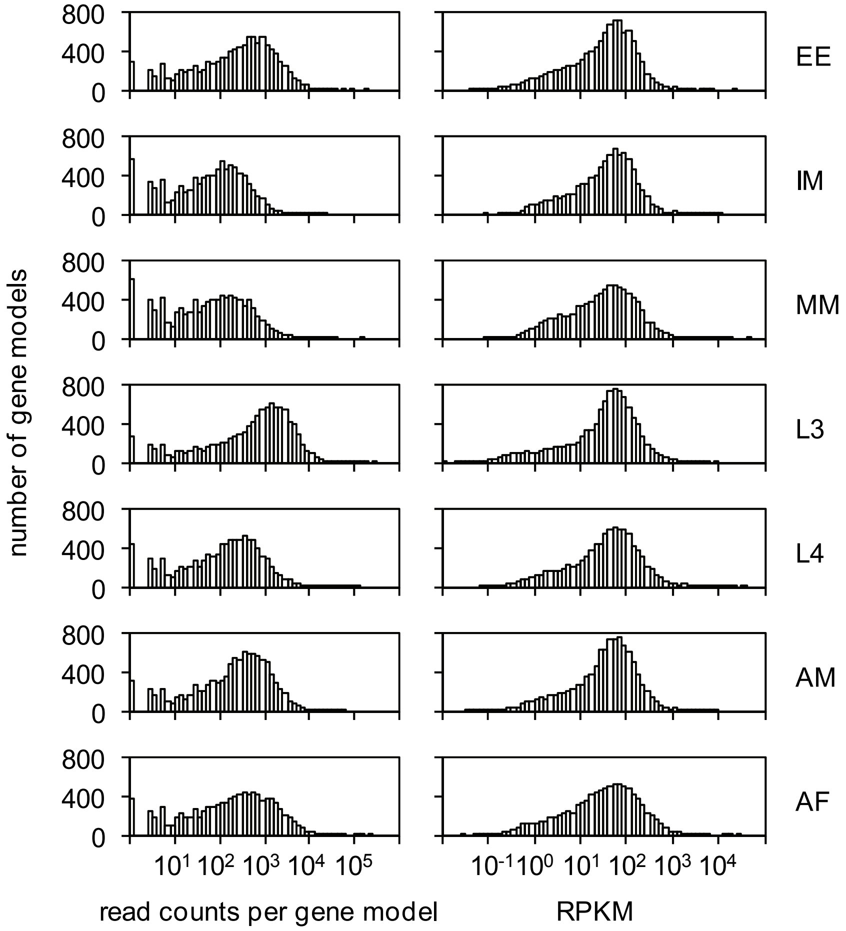

Supplement: Figure S1 — Histogram of raw read counts per gene model and scaling-normalized RPKM values. To facilitate transcript abundance comparisons between genes and stages, read counts were RPKM-transformed and TMM-normalized [9], [10]. The distribution of transcript level estimates indicated 4 to 5 logs of dynamic range. (PDF) [file pntd.0001409.s001.pdf]
