# Supplementary material for: A Deep Sequencing Approach to Comparatively Analyze the Transcriptome of Lifecycle Stages of the Filarial Worm, Brugia malayi
Source: PLoS Negl Trop Dis. 2011 Dec 13;5(12):e1409. doi: 10.1371/journal.pntd.0001409 (PMC3236722; doi:10.1371/journal.pntd.0001409)

Figure S2. Genome-wide distribution of the dispersion parameter estimating stage-to-stage variability in transcript abundance. Based on the NB dispersion parameter, genes were ordered from the most variable to the least variable, and partitioned into four equal-sized groups as indicated by the horizontal dotted lines. GO term enrichment tests were performed on each of the four groups (Table S2).

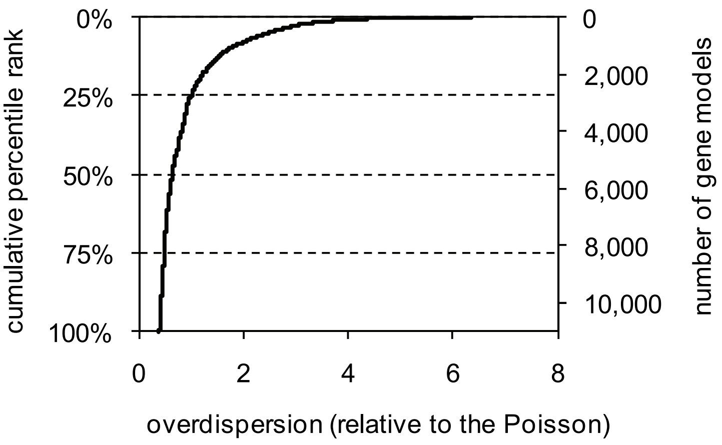

Supplement: Figure S2 — Genome-wide distribution of the dispersion parameter estimating stage-to-stage variability in transcript abundance. Based on the NB dispersion parameter, genes were ordered from the most variable to the least variable, and partitioned into four equal-sized groups as indicated by the horizontal dotted lines. GO term enrichment tests were performed on each of the four groups (Table S2). (PDF) [file pntd.0001409.s002.pdf]
