# Supplementary material for: A Deep Sequencing Approach to Comparatively Analyze the Transcriptome of Lifecycle Stages of the Filarial Worm, Brugia malayi
Source: PLoS Negl Trop Dis. 2011 Dec 13;5(12):e1409. doi: 10.1371/journal.pntd.0001409 (PMC3236722; doi:10.1371/journal.pntd.0001409)

Figure S3. Venn diagram analysis to identify genes with female somatic tissue- or germline-enriched expression pattern. (1) genes with enriched expression in adult female relative to eggs & embryos; (2) genes with enriched expression in eggs & embryos relative to adult male; (3) genes with enriched expression in adult female and/or eggs & embryos relative to all other stages.

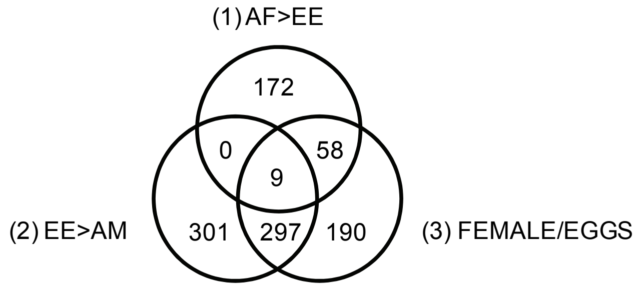

Supplement: Figure S3 — Venn diagram analysis to identify genes with female somatic tissue- or germline-enriched expression pattern. (1) genes with enriched expression in adult female relative to eggs & embryos; (2) genes with enriched expression in eggs & embryos relative to adult male; (3) genes with enriched expression in adult female and/or eggs & embryos relative to all other stages. (PDF) [file pntd.0001409.s003.pdf]
