# Supplementary material for: A Deep Sequencing Approach to Comparatively Analyze the Transcriptome of Lifecycle Stages of the Filarial Worm, Brugia malayi
Source: PLoS Negl Trop Dis. 2011 Dec 13;5(12):e1409. doi: 10.1371/journal.pntd.0001409 (PMC3236722; doi:10.1371/journal.pntd.0001409)
Supplement: Table S1 — Total number of reads sequenced and mapped to the genome. (PDF) [file pntd.0001409.s004.pdf]

Table S1. Total number of reads sequenced and mapped to the genome.

library	total sequenced	unambiguously mapped to	
		gene models	unannotated regions
eggs & embryos	13,522,277	7,549,405	1,068,846
immature MF	11,829,651	1,614,177	178,388
mature MF	7,979,044	2,393,380	163,257
L3	27,021,114	18,143,064	2,003,074
L4	14,467,007	5,055,440	282,023
adult male	10,987,792	5,957,677	502,938
adult female	18,345,242	7,884,405	502,388
	104,152,127	48,597,548	4,700,914
