# Supplementary material for: A Deep Sequencing Approach to Comparatively Analyze the Transcriptome of Lifecycle Stages of the Filarial Worm, Brugia malayi
Source: PLoS Negl Trop Dis. 2011 Dec 13;5(12):e1409. doi: 10.1371/journal.pntd.0001409 (PMC3236722; doi:10.1371/journal.pntd.0001409)
Supplement: Table S2 — List of over-represented GO terms in groups of genes with different levels of overall variability in their transcript abundance across lifecycle stages. Genes were partitioned into four equal-sized groups designated as Q1 to 4 in the order of decreasing variability (Figure S2). Over-represented GO terms with p-value<0.01 were included. (PDF) [file pntd.0001409.s005.pdf]

Table S2. List of over-represented GO terms in groups of genes with different levels of overall variability in their transcript abundance across lifecycle stages. Genes were partitioned into four equal-sized groups designated as Q1 to 4 in the order of decreasing variability (Figure S2). Over-represented GO terms with p-value < 0.01 were included.

Q1	Q2	Q3	Q4	GO terms	
				**cellular component**	
		6.5E-12	9.1E-04	ribosome	GO:0005840
		6.9E-05		ribonucleoprotein complex	GO:0030529
			4.4E-07	cytoplasm	GO:0005737
			3.1E-05	proteasome complex	GO:0000502
			6.3E-04	mitochondrial inner membrane	GO:0005743
			9.2E-04	proteasome core complex	GO:0005839
				**molecular function**	
4.8E-12				structural constituent of cuticle	GO:0042302
1.4E-10				serine-type endopeptidase inhibitor activity	GO:0004867
4.4E-06				phosphoprotein phosphatase activity	GO:0004721
2.3E-05				structural molecule activity	GO:0005198
5.8E-05				cysteine-type peptidase activity	GO:0008234
8.7E-05				protein kinase activity	GO:0004672
2.0E-04				hedgehog receptor activity	GO:0008158
2.3E-04				protein tyrosine kinase activity	GO:0004713
7.2E-04				extracellular matrix structural constituent	GO:0005201
	4.8E-06			RNA-directed DNA polymerase activity	GO:0003964
		3.8E-11		structural constituent of ribosome	GO:0003735
			8.4E-10	binding	GO:0005488
			3.6E-09	ligase activity	GO:0016874
			1.3E-06	ATP binding	GO:0005524
			5.9E-06	aminoacyl-tRNA ligase activity	GO:0004812
			9.7E-06	protein transporter activity	GO:0008565
			4.1E-05	4 iron, 4 sulfur cluster binding	GO:0051539
			5.2E-05	nucleotide binding	GO:0000166
			5.8E-05	acid-amino acid ligase activity	GO:0016881
			1.3E-04	unfolded protein binding	GO:0051082
			2.5E-04	heat shock protein binding	GO:0031072
			6.6E-04	helicase activity	GO:0004386
			8.3E-04	endopeptidase activity	GO:0004175
			9.2E-04	threonine-type endopeptidase activity	GO:0004298
				**biological process**	
1.0E-04				protein amino acid phosphorylation	GO:0006468
	1.6E-07			DNA integration	GO:0015074
	1.3E-05			RNA-dependent DNA replication	GO:0006278
		1.6E-07	1.3E-06	translation	GO:0006412
			1.9E-07	protein transport	GO:0015031
			7.4E-07	intracellular protein transport	GO:0006886
			8.9E-07	protein folding	GO:0006457
			7.3E-05	protein modification process	GO:0006464
			9.9E-05	tRNA aminoacylation for protein translation	GO:0006418
			5.1E-04	ubiquitin-dependent protein catabolic process	GO:0006511
			5.1E-04	protein catabolic process	GO:0030163
			5.2E-04	regulation of actin filament polymerization	GO:0030833
			5.8E-04	vesicle-mediated transport	GO:0016192
			9.2E-04	proteolysis involved in cellular protein catabolic process	GO:0051603
