# Supplementary material for: A Deep Sequencing Approach to Comparatively Analyze the Transcriptome of Lifecycle Stages of the Filarial Worm, Brugia malayi
Source: PLoS Negl Trop Dis. 2011 Dec 13;5(12):e1409. doi: 10.1371/journal.pntd.0001409 (PMC3236722; doi:10.1371/journal.pntd.0001409)
Supplement: Table S3 — List of over-represented GO terms and their corresponding p-values among genes that show relative transcriptional enrichment in pairwise comparisons (Figure 3D). Female somatic tissue- and germiline-enriched patterns were defined using Venn diagram analysis (Figure S3). (PDF) [file pntd.0001409.s006.pdf]

Table S3. List of over-represented GO terms and their corresponding p-values among genes that show relative transcriptional enrichment in pairwise comparisons (Figure 3D). Female somatic tissue- and germiline-enriched patterns were defined using Venn diagram analysis (Figure S3).

Pairwise comparisons				GO terms	
(1)	AM	1.2E-03	C	cytoskeleton	GO:0005856
		5.2E-03	C	prefoldin complex	GO:0016272
		1.7E-12	F	structural molecule activity	GO:0005198
		2.2E-11	F	phosphoprotein phosphatase activity	GO:0004721
		3.6E-07	F	protein kinase activity	GO:0004672
		2.3E-06	F	protein tyrosine phosphatase activity	GO:0004725
		1.3E-05	F	phosphatase activity	GO:0016791
		1.3E-04	F	kinase activity	GO:0016301
		2.8E-03	F	protein tyrosine kinase activity	GO:0004713
		1.3E-06	P	protein amino acid phosphorylation	GO:0006468
		3.6E-06	P	protein amino acid dephosphorylation	GO:0006470
		1.6E-05	P	dephosphorylation	GO:0016311
		5.2E-03	P	cell differentiation	GO:0030154
	AF	5.4E-17	C	nucleus	GO:0005634
		5.6E-30	F	transcription factor activity	GO:0003700
		1.5E-25	F	sequence-specific DNA binding	GO:0043565
		3.5E-14	F	DNA binding	GO:0003677
		5.0E-11	F	ligand-dependent nuclear receptor activity	GO:0004879
		7.8E-10	F	steroid hormone receptor activity	GO:0003707
		4.3E-05	F	receptor activity	GO:0004872
		5.7E-04	F	transcription regulator activity	GO:0030528
		2.7E-03	F	transcription cofactor activity	GO:0003712
		4.7E-03	F	protein dimerization activity	GO:0046983
		6.6E-03	F	zinc ion binding	GO:0008270
		2.6E-27	P	regulation of transcription, DNA-dependent	GO:0006355
		1.1E-18	P	regulation of transcription	GO:0045449
		4.6E-09	P	transcription	GO:0006350
		4.2E-03	P	multicellular organismal development	GO:0007275
		6.6E-03	P	homophilic cell adhesion	GO:0007156
		8.5E-03	P	cell-matrix adhesion	GO:0007160
(2)	somatic tissue	1.8E-04	F	voltage-gated chloride channel activity	GO:0005247
		4.9E-04	F	structural constituent of cuticle	GO:0042302
		7.8E-03	F	lipid binding	GO:0008289
		1.8E-04	P	chloride transport	GO:0006821
		7.6E-03	P	proteolysis	GO:0006508
	germline tissue	5.0E-15	C	nucleus	GO:0005634
		1.0E-03	C	plasma membrane	GO:0005886
		8.4E-03	C	proteinaceous extracellular matrix	GO:0005578
		1.9E-28	F	transcription factor activity	GO:0003700
		1.4E-26	F	sequence-specific DNA binding	GO:0043565
		3.1E-15	F	DNA binding	GO:0003677
		3.0E-10	F	transcription regulator activity	GO:0030528
		5.7E-09	F	ligand-dependent nuclear receptor activity	GO:0004879
		5.9E-07	F	steroid hormone receptor activity	GO:0003707
		2.8E-06	F	serine-type endopeptidase inhibitor activity	GO:0004867
		1.4E-05	F	structural constituent of cuticle	GO:0042302

		1.5E-05	F	receptor activity	GO:0004872
		1.1E-04	F	calcium ion binding	GO:0005509
		1.2E-03	F	metallopeptidase activity	GO:0008237
		2.2E-03	F	protein dimerization activity	GO:0046983
		1.1E-25	P	regulation of transcription, DNA-dependent	GO:0006355
		5.0E-25	P	regulation of transcription	GO:0045449
		9.0E-10	P	transcription	GO:0006350
		7.1E-06	P	homophilic cell adhesion	GO:0007156
		3.1E-05	P	multicellular organismal development	GO:0007275
		2.3E-03	P	cell adhesion	GO:0007155
		5.9E-03	P	Wnt receptor signaling pathway	GO:0016055
(3)	IM	8.0E-03	C	proton-transporting ATP synthase complex, coupling factor F(o)	GO:0045263
		1.8E-03	F	asparagine-tRNA ligase activity	GO:0004816
		2.5E-03	F	hydrogen ion transporting ATP synthase activity, rotational mechanism	GO:0046933
		7.4E-03	F	exodeoxyribonuclease III activity	GO:0008853
		9.4E-03	F	cytidine deaminase activity	GO:0004126
		1.8E-03	P	asparaginyl-tRNA aminoacylation	GO:0006421
		9.4E-03	P	cytidine metabolic process	GO:0046087
	MM	1.8E-05	C	cytoskeleton	GO:0005856
		5.8E-04	F	structural molecule activity	GO:0005198
(4)	L3	5.4E-03	C	membrane	GO:0016020
		2.0E-08	F	cysteine-type peptidase activity	GO:0008234
		1.3E-07	F	cysteine-type endopeptidase activity	GO:0004197
		5.7E-04	F	serine-type endopeptidase inhibitor activity	GO:0004867
		3.0E-03	F	magnesium ion binding	GO:0000287
		3.5E-03	F	hedgehog receptor activity	GO:0008158
		5.8E-03	F	oxidoreductase activity	GO:0016491
		8.6E-03	F	oxidoreductase activity,<br>acting on the CH-OH group of donors, NAD or NADP as acceptor	GO:0016616
		8.6E-03	F	ion channel activity	GO:0005216
		9.8E-04	P	proteolysis	GO:0006508
		2.3E-03	P	oxidation reduction	GO:0055114
		9.6E-03	P	metabolic process	GO:0008152
	L4	1.8E-08	F	structural constituent of cuticle	GO:0042302
		1.8E-03	P	cellular component organization	GO:0016043
		7.5E-03	P	response to oxidative stress	GO:0006979
		9.5E-03	P	regulation of transcription, DNA-dependent	GO:0006355
